# Biomanufacturing Recombinantly Expressed Cripto-1 Protein in Anchorage-Dependent Mammalian Cells Growing in Suspension Bioreactors within a Three-Dimensional Hydrogel Microcarrier

**DOI:** 10.3390/gels9030243

**Published:** 2023-03-18

**Authors:** Rachel Lev, Orit Bar-Am, Yoni Lati, Ombretta Guardiola, Gabriella Minchiotti, Dror Seliktar

**Affiliations:** 1 Faculty of Biomedical Engineering, Technion—Israel Institute of Technology, Haifa 3200003, Israel; 2 The Norman Seiden Multidisciplinary Graduate Program in Nanoscience and Nanotechnology, The Russell Berrie Nanotechnology Institute, Technion—Israel Institute of Technology, Haifa 3200003, Israel; 3 Stem Cell Fate Laboratory, Institute of Genetics and Biophysics “A. Buzzati Traverso”, CNR, 80131 Naples, Italy

**Keywords:** hydrogel, recombinant protein, bioreactor, microcarrier, HEK293 cells, protein therapeutics

## Abstract

Biotherapeutic soluble proteins that are recombinantly expressed in mammalian cells can pose a challenge when biomanufacturing in three-dimensional (3D) suspension culture systems. Herein, we tested a 3D hydrogel microcarrier for a suspension culture of HEK293 cells overexpressing recombinant Cripto-1 protein. Cripto-1 is an extracellular protein that is involved in developmental processes and has recently been reported to have therapeutic effects in alleviating muscle injury and diseases by regulating muscle regeneration through satellite cell progression toward the myogenic lineage. Cripto-overexpressing HEK293 cell lines were cultured in microcarriers made from poly (ethylene glycol)-fibrinogen (PF) hydrogels, which provided the 3D substrate for cell growth and protein production in stirred bioreactors. The PF microcarriers were designed with sufficient strength to resist hydrodynamic deterioration and biodegradation associated with suspension culture in stirred bioreactors for up to 21 days. The yield of purified Cripto-1 obtained using the 3D PF microcarriers was significantly higher than that obtained with a two-dimensional (2D) culture system. The bioactivity of the 3D-produced Cripto-1 was equivalent to commercially available Cripto-1 in terms of an ELISA binding assay, a muscle cell proliferation assay, and a myogenic differentiation assay. Taken together, these data indicate that 3D microcarriers made from PF can be combined with mammalian cell expression systems to improve the biomanufacturing of protein-based therapeutics for muscle injuries.

## 1. Introduction

The production of recombinant therapeutic proteins has become increasingly popular for the large-scale manufacturing of medical-grade biotherapeutics [1,2,3]. Although *Escherichia coli* (*E. coli*) and yeast are the most established organisms for biomanufacturing of recombinant proteins, production in mammalian cells has considerable advantages, particularly for complex protein products [3,4]. When produced in mammalian cells, recombinant human proteins undergo more accurate post-translational processing, which is crucial for proteins that require complicated tertiary structures to exhibit biological activity [3,5,6,7]. Compared to the simpler expression systems of *E. coli* and yeast, one of the biggest drawbacks of using mammalian cells can be the more stringent culture conditions, which can also involve anchorage-dependent growth with less efficient two-dimensional (2D) cultures. The 2D culture systems are reliable and well defined, but the limited growth surface area places them at a disadvantage with respect to large-scale production. Over the past two decades, much effort has been directed at adapting mammalian cell expression systems for more efficient three-dimensional (3D) culture in suspension. This has been achieved by either adapting the mammalian cells to continuous cell lines (CCLs), such as with Chinese hamster ovary (CHO) cells that can grow directly in suspension, or growing the mammalian cells with microcarriers [8,9,10]. Whereas certain mammalian cell types that are anchorage-dependent may not be able to conform to suspension culture, most anchorage-dependent cells can grow in or on microcarriers.

The manufacturing capacity of systems using anchorage-dependent mammalian cells has become a critical issue in the development of protein-based therapeutics. As new therapeutic proteins produced in these cells are introduced, 2D systems are initially used for making high-quality products with sufficient production yields for achieving the research and development goals. As the biotherapeutic reaches the clinical phases of development, efficient bioprocessing using large-scale, state-of-the-art manufacturing paradigms becomes essential. The bioprocessing of these biotherapeutics must also meet stringent commercial and clinical requirements, and the 2D manufacturing paradigms initially applied substantially limit the potential to reach the high production yields required by industry for more advanced stages of clinical development. Therefore, as new therapeutic proteins progress toward the clinic, production efficiency must be addressed together with clinical efficacy—usually with the help of the microcarrier systems. In this context, microcarriers combined with suspension bioreactors address many of the key manufacturing drawbacks of the 2D production methodologies. Although conventional microcarriers provide a higher surface area for anchorage-dependent cell cultivation, cells that grow on these polymeric systems exhibit 2D growth characteristics. Therefore, the potential of 3D suspension cultivation using microcarriers may not be fully realized until microcarriers can accommodate cell growth within the volume of the carrier [11,12]. Indeed, many new cell carrier systems for protein production are premised on porous polymeric scaffolds or hydrogel scaffolds that can accommodate 3D cell growth in the pores or bulk of the material. The polymeric materials can be designed as spherical or disk-shaped microcarriers with increased cell growth capacity and the advantages of high surface area to volume ratios.

Several research groups worldwide have developed and optimized the production of therapeutic proteins in stirred suspension bioreactors utilizing 2D and 3D microcarrier-based cultivation systems [1,13,14]. Various types of microcarriers have been examined for their ability to serve as growth substrates for cell cultivation in a bioreactor, including solid and porous materials. Solid microcarriers that support 2D cell attachment on their surface are typically made from synthetic materials such as glass or crosslinked polystyrene, and these can be further modified with an electrical charge potential or immobilized proteins for better cell adhesion [15]. Macroporous materials that support 3D cell culture based on a sponge-like architecture are typically made from dextran, collagen, or alginate. Microporous hydrogels can also be used as microcarriers for 3D cell culture in suspension by providing cells with an encapsulating milieu [16,17,18]. In this approach, the microporous hydrogels entrap the cells in the bulk of the materials during the hydrogel gelation process [19,20,21]. This requires a bioprocessing step that can accommodate the need to procure large quantities of cell-laden hydrogel microcarriers prior to the protein production run. A number of techniques are available for this bioprocessing, including microfluidics, emulsion polymerization strategies, drop-wise polymerization, or polymerization on superhydrophobic surfaces [22,23,24,25,26,27].

A 3D microcarrier-based cultivation in stirred suspension bioreactors offers pronounced advantages over the use of 2D microcarriers, which are limited by surface area and hampered by hydrodynamic or collisional forces associated with stirred suspension bioreactors [28,29,30]. Because the 3D microcarriers made from encapsulating microporous hydrogels take advantage of the volume of the microcarrier for cell growth, the cells growing in the bulk of the hydrogel matrix are also protected from the mechanical stress environment of the bioreactor. This combined advantage allows long-term, semi-continuous culturing which contributes to greater biomass production capacity and scalability [31,32,33]. This advantage is also particularly important for secreted proteins that can diffuse through the hydrogel matrix. Specifically, cells expressing proteins that are secreted into the 3D microporous hydrogel system can be periodically harvested from the culture medium without ever disturbing the cells inside the hydrogel matrix. These advantages can bring 3D hydrogel microcarriers closer to automatization, which is a requirement of the biotechnology industry [28,34,35].

Spherical microgels made from hydrogel biomaterials represent a promising class of microcarriers with the potential to be used in 3D cell cultivation systems [9,36,37]. These microcarriers are receiving more widespread attention owing to their viscoelastic behavior that mimics that of the natural environment of the extracellular matrix (ECM) of anchorage-dependent cells [38]. Hydrogels are classified as either natural, synthetic, or semisynthetic. Naturally derived hydrogels provide adhesion sites, which support cell function; however, they lack the mechanical properties necessary for long-term cultivation in suspension cultures. Synthetic hydrogels are reproducible and exhibit stable mechanical properties that can be easily manipulated but are deficient in bioactive functionality, such as possessing adhesive sites for cell attachment. Semisynthetic hydrogels, which contain both biological and synthetic elements, constitute the optimal combination, making them amenable for suspension culture applications. Specifically, these hydrogels possess stable mechanical properties owing to their synthetic composition and can exhibit cell adhesivity and proteolytic degradability because of their biological domains [39,40,41].

The biological and synthetic composition of a semisynthetic hydrogel used for making microcarriers will depend on the type of cells used for protein production, as well as the requirements of the suspension bioreactor system. Polyethylene glycol (PEG)-based hydrogels have been used extensively in 3D cultures of various cell types for tissue engineering; they exhibit high biocompatibility as well as versatile physical characteristics depending on their weight percent, molecular chain length, and crosslinking density [42]. The PEG component can maintain the structural integrity of the composite structure, thereby ensuring microcarrier stability under the hydrodynamic loads of suspension bioreactors. Conjugating PEG with fibrinogen, the natural substrate for tissue remodeling, provides bioactivity to the PEG hydrogels, which would otherwise be lacking [42]. The gelation process of PEG-fibrinogen (PF) is mild and nontoxic for cells, even when cells are suspended in the precursor solution [43]. The natural cell adhesion motifs and protease cleavage sites on fibrinogen facilitate the stable maintenance of cells while simultaneously allowing dynamic changes such as cell motility and invasion, which are necessary for cell growth and proliferation [44]. These features of PF hydrogels, including biological motifs, chemical crosslinking, and ECM-like physical properties, make them an effective candidate microcarrier material for working with anchorage-dependent cells under the hydrodynamics of bioreactors [42].

PF hydrogels have been used to encapsulate various kinds of mammalian cells, such as sheep aortic smooth muscle cells (SASMCs), human foreskin fibroblasts (HFFs), and human embryonic kidney 293 (HEK293) cells [21,44,45]. The latter, when encapsulated in PF hydrogels, have managed to maintain high cell viability for long durations. This strategy was effective in facilitating the production of a model protein, acetylcholine esterase (AChE), to enhance production capacity several-fold over 2D cultures. Using PF hydrogels as a microcarrier for HEK293 cells and cultivating them in stirred suspension bioreactors led to a higher yield of AChE. It was further found that the PF microcarriers provided a biocompatible environment for cell culture, as indicated by a high percentage of living cells and the formation of cell clusters in the 3D PF microcarriers over the culture duration [21].

In the present study, we aimed to scale up the production of a therapeutic protein called Cripto-1 using recombinantly modified HEK293 cells grown in PF microcarriers. Cripto-1 is a 27 kDa membrane glycosylphosphatidylinositol (GPI)-anchored protein, which belongs to the EGF–CFC protein family and plays an important regulatory role in embryonic development [46]. It can act both as a ligand via the Nodal/Alk4-independent signaling pathway and as a co-receptor for Nodal through activation of the ALK4/ActRIIB receptor complex [5,47,48]. Previous findings have indicated that Cripto-1, which is expressed in myoblasts of regenerative muscles but not in normal muscle fibers, influences myostatin signaling in myoblasts [49,50]. Guardiola et al. showed that Cripto-1 modulates myogenic cell determination and promotes proliferation by antagonizing myostatin. In addition, myostatin and Cripto-1 are expressed in regenerating muscles, and the latter attenuates the myostatin signaling pathway. They also demonstrated that Cripto-1 antagonizes the antiproliferative effect of myostatin on isolated myofibers, promoting myogenic commitment, and simultaneously blocks myostatin activity, promoting the entry of satellite cells into S phase and their commitment to differentiation. The promising results of their study suggest that Cripto-1 is a novel regulator of muscle regeneration and satellite cell progression toward the myogenic lineage [51].

The availability of large quantities of biologically activated Cripto-1 is crucial to the advancement of clinical studies using this promising therapeutic protein. Recombinant Cripto-1 protein cannot be expressed in *E. coli*, yeast, or CHO cells because of a mutation of the Asn63 residue that prevents post-translational modification (i.e., glycosylation), which affects protein activity in vivo [5]. Hence, our goal was to provide Cripto-1-overexpressing HEK293 cells with optimal conditions in a 3D culture suspension bioreactor, including a supportive microenvironment for improved cell viability leading to enhanced protein yields. For this purpose, PF microcarriers were designed to enable the HEK293 cell survival, proliferation, and secretion of large quantities of the recombinantly expressed therapeutic protein. Cripto-1-overexpressing HEK293 cell lines transfected with His-tagged protein were encapsulated in PF microcarriers and cultivated in stirred suspension bioreactors. The PF microcarriers were designed to possess high mechanical strength to resist the shear forces and biodegradation associated with long-term suspension culture in the bioreactors. The yield of Cripto-1 protein obtained using this system was significantly higher than that obtained with the traditional 2D system. Cripto-1 bioactivity was maintained throughout the production and purification process. Moreover, long-term evaluation showed that the stability and integrity of Cripto-1 were maintained for up to 6 years after production in the PF microcarrier system.

## 2. Results

### 2.1. Production of Recombinant Cripto in a 3D PF Microcarrier-Based System Using Stirred Suspension Bioreactors

**Figure 1:**
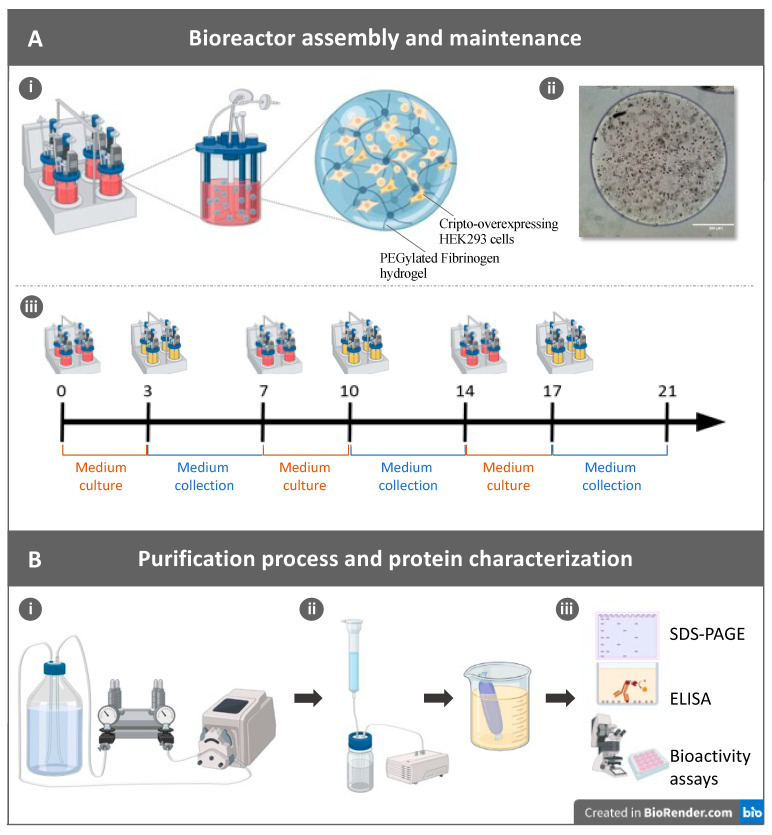
Schematic illustration of PF microcarriers and the bioprocessing procedure for making Cripto protein. (**A**) **i**: A set of bioreactors were used for Cripto production; each bioreactor cultivates microcarriers made of PEG-fibrinogen (PF) hydrogel containing Cripto-overexpressing HEK293 cells. **ii**: Phase contrast microscopy image of a typical microcarrier shows individual cells growing in the 3D PF hydrogel. **iii**: Bioreactor cultivation timeline of a 3-week culture period of the microcarriers in the bioreactors. Cells were cultured in growth medium (depicted in red) on days 0, 7, and 14 for 3 days of maintenance, with the medium then changed for starvation medium (depicted in yellow) on days 3, 10, and 17 for the collection step that lasted 4 days each time. (**B**) **i**: The manufactured Cripto protein was concentrated and diafiltrated using PALL Centramate membrane. **ii**: The Cripto protein was purified using a Ni-NTA column followed by a dialysis step. **iii**: Finally, structural and functional characterization of the purified Cripto protein was performed by SDS-PAGE, ELISA, and bioactivity assays.

**Figure 2:**
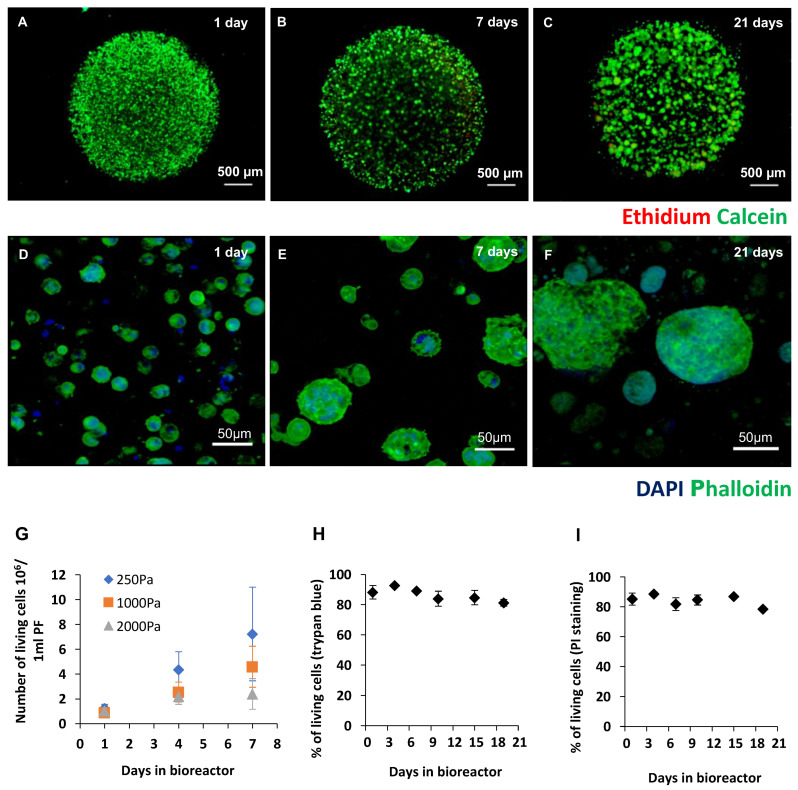
Viability of HEK293 cells within PF microcarriers in 3D culture. (**A**–**C**) Cell viability of HEK293 cells growing in the 3D PF microcarriers (8 mg/mL, G’ = 250–2000 Pa) in bioreactors was verified using a calcein/ethidium assay. Initially, viable cells were distributed uniformly in the microcarriers on day 1 (**A**) and organized into colonies by day 7 (**B**), remaining viable beyond day 21 (**C**). High-magnification images of cells stained with f-actin FITC-phalloidin (green) and DAPI (nucleus in blue) show the cells initially distributed uniformly within the microcarriers on day 1 (**D**). After 7 and 21 days, the cell colonies were also distributed uniformly throughout the microcarrier (**E**,**F**). (**G**) The number of living cells was quantified in the microcarriers as a function of culture time (up to 7 days) and PF modulus (G’ = 250–2000 Pa). The G’ values shown in the graph correspond to the PF hydrogels made from different formulations, as determined based on data provided in Supplementary Figure S1. As can be seen, both PF modulus and culture time affected the number of HEK293 cells in the microcarriers. (**H**) Cell viability over 21 days in PF microcarriers (G’ = 1000) with suspension culture, as measured by a trypan blue exclusion assay. Each time point represents data from an average of several bioreactors (3 < *n* < 18). (**I**) Cell proliferation in PF microcarriers (G’ = 1000) with suspension culture in bioreactors for up to 21 days, as measured by a PI staining assay; each time point is represented by an average of at least three different experiments (*n* > 3). All results shown are as mean ± S.D. of at least three independent experiments.

Cripto-overexpressing HEK293 cell lines transfected with a soluble form of His-tagged Cripto-1 protein were encapsulated in the PF microcarriers made from PF and different concentrations of PEG-DA. Three PF formulations were initially tested using 8 mg/mL PF and the addition of 0%, 1%, and 2% PEG-DA to reach a final shear storage modulus of approximately 250 Pa, 1000 Pa, and 2000 Pa, respectively. The 8 mg/mL PF concentration that was used in this study was based on our previous experiments using this hydrogel system for 3D HEK293 cell cultures [21]. The microcarriers were prepared using a droplet-based formation of PF precursor solution mixed with ~8.5 × 10^6^ cells/mL, deposited on a superhydrophobic surface, and exposed to UV light to initiate a photo-polymerization reaction with the PF. The cells were cultivated within the microcarriers in stirred suspension bioreactors (Figure 1). The live/dead staining of the microcarriers showed individual cells encapsulated within the PF matrix after one day forming colonies of viable cells within the matrix after 7 and 21 days (Figure 2A–C). High-magnification live/dead images for day 1 and day 7 are shown in the supplementary data (Supplementary Figure S5). FITC-rhodamine F-actin staining of the microcarriers showed the progression from single cells dispersed within the PF matrix at day 1 to multicellular colonies at day 7 and day 21 (Figure 2D–F). The initially rounded cells remained rounded in the PF matrix throughout the culture duration, and the proliferating cells formed colonies within the matrix during this time. High-magnification confocal images of an individual cell colony after 21 days with TRITC-rhodamine staining for f-actin and a SYTOX-green nuclear stain underscore the consequence of the PF matrix’s confining effects on cell proliferation and colony formation (Supplementary Figure S6).

The number of living cells in the microcarriers was quantified and shown to increase relative to the culture duration and the mechanical properties of the PF matrix (Figure 2G). Based on these initial assessments of viability, we decided to use PF microcarriers made from 8 mg/mL PF and 1% PEG-DA (G’ = 1000 Pa) in all further experiments. Accordingly, cells cultured in these microcarriers in suspension for up to three weeks were quantitatively evaluated for viability during the duration of the Cripto-1 production cycle (Figure 2H,I). The quantitative viability data of the HEK293 cells within microcarriers show consistent levels of around 80% throughout the 21 days in culture, as measured by both trypan blue and PI incorporation assays (Figure 2H,I).

**Figure 3:**
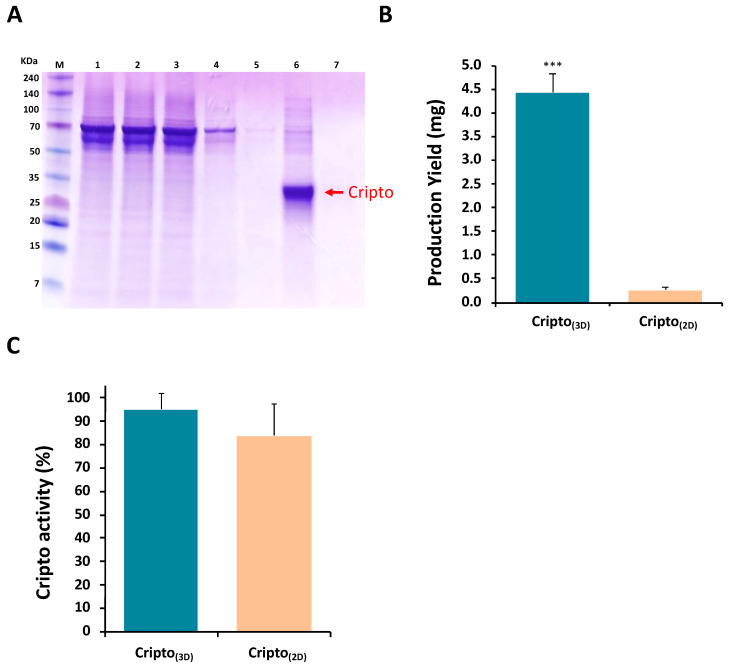
The production yield, activity, and purification of recombinant Cripto produced in the 3D microcarriers as compared to the 2D method. (**A**) SDS-PAGE analysis of the purification steps of recombinant Cripto: lane M is the protein molecular weight marker; lane 1 is the protein solution from ultrafiltration; lane 2 is the flowthrough after the first passage through the His-tag affinity Ni-NTA resin; lane 3 is the flowthrough after the second passage through the same resin; lane 4 is the first wash step; lane 5 is the second wash step; lane 6 is the first elution from the His-tag affinity Ni-NTA resin; and lane 7 is the second elution from the same resin. The band at approximately 27 kDa is the Cripto protein (indicated by the red arrow). (**B**) Quantitative amounts of Cripto protein produced in the 3D batch (Cripto_(3D)_) with HEK293 cells encapsulated in PF microcarriers and incubated in bioreactors after three rounds of harvesting are compared to the maximum amount of Cripto produced in the 2D batch (Cripto_(2D)_) with HEK293 cells adherent to cell culture plates and cultured to their density threshold limits. An initial cell seeding of 3.2 × 10^6^ cells was used for both techniques. (**C**) The biological activity of recombinant Cripto was compared for Cripto produced in PF microcarriers versus the 2D method by measuring binding affinity to the AlK4 receptor. Four independent experiments were carried out for the 3D system and three independent experiments were performed for the 2D cultivation method. Results are shown as mean ± S.D. *** indicates *p* < 0.001.

The cell-laden microcarriers were used for the Cripto production cycle as illustrated in Figure 1. After an initial 3 days of incubation in growth medium, starvation medium was used to collect the secreted Cripto protein on a daily basis for 4 days, and this cycle was repeated three times (Figure 1A). The collected Cripto was defrosted and pooled for ultrafiltration followed by His-tag affinity chromatography and dialysis (Figure 1B). SDS-PAGE results of the Cripto protein in the fractions of the chromatography obtained at different stages of the process confirmed the presence of the Cripto, as well as the effects of the purification steps (Figure 3A). Seven fractions were collected in total, including the concentrated sample after ultrafiltration, samples from the first and second flowthrough of the affinity chromatography run, two samples after each wash, and two samples eluted from the Ni-NTA resin. A band corresponding to Cripto-1 protein was clearly visible at 27 kDa after elution from the Ni-NTA resin [5] (Figure 3A arrow). Cripto-1 purification on the Ni-NTA column was also verified by the gradual disappearance of the dominant protein bands at around 70 kDa during the chromatography process (Figure 3A). These bands are likely attributed to residues of fibrinogen chains originating from the PF microcarrier-based cultivation system.

The production yields of purified Cripto-1 protein obtained from the 3D PF microcarrier system (Cripto_(3D)_) were quantitively compared to the yields obtained from traditional 2D culture production (Cripto_(2D)_). The comparative experiments were performed using the same initial number of cells in each culture system. A total of six independent experiments were performed using a seeding of 3.2 × 10^6^ HEK293 cells for each treatment (i.e., 3D versus 2D). The experiments using the 3D system were conducted as per the protocol illustrated in Figure 1, whereas the experiments using the 2D cultivation method were conducted on TCP dishes until the cells approached a density threshold and started to detach from the plate. The concentration of total purified Cripto-1 from all experiments was determined by means of an ELISA. The average production yield of the 3D method (4.5 mg Cripto) was more than one order of magnitude higher than that of the 2D method (0.25 mg Cripto) (Figure 3B). These data represent the amount of protein that was produced with each technique using the fixed initial cell population. The Cripto-1 produced in 3D microcarriers and 2D flasks was also tested for its biological activity using an ELISA kit that assesses its ability to bind to the AlK4 receptor. The binding level of Cripto-1 protein produced by the 3D microcarriers was 93%, while the binding level of Cripto-1 produced by the 2D system was 84% (Figure 3C); however, there was no statistically significant difference between the two systems in terms of binding levels as measured by the AlK4 receptor ELISA.

### 2.2. In Vitro Functional Activity of Purified Recombinant Cripto

**Figure 4:**
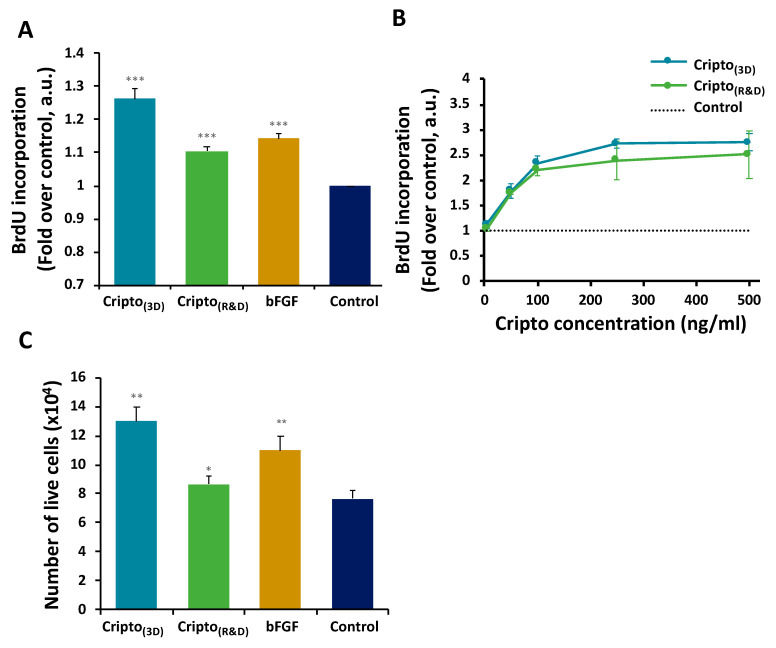
The effect of Cripto produced in 3D microcarriers on C2C12 cell proliferation detected by the BrdU incorporation assay. (**A**) C2C12 myoblast proliferation was evaluated by the BrdU incorporation assay after being cultured for 48 h in serum-free medium containing Cripto produced in 3D microcarriers (Cripto_(3D)_) or commercially available Cripto (Cripto_(R&D)_). Also evaluated were a bFGF medium positive control and a serum-free medium negative control. (**B**) The recombinant Cripto induces myoblast proliferation in a dose-dependent pattern; increasing concentrations of Cripto_(3D)_ and Cripto_(R&D)_ were added to C2C12 cells, and proliferation was quantified by the BrdU incorporation assay. (**C**) Cell proliferation was further evaluated by counting the total number of live cells. The proliferative effect of Cripto_(3D)_ was compared with that of commercial Cripto_(R&D)_. The data are presented as mean ± S.D. from at least three independent experiments. * indicates *p* < 0.05, ** indicates *p* < 0.01, *** indicates *p* < 0.001.

To demonstrate the proliferative effect of the recombinantly produced Cripto-1 on myoblasts, a BrdU incorporation assay was performed alongside cell counting experiments. C2C12 myoblasts were cultured for 48 h in starvation medium (0.5% FBS) containing either the Cripto-1 produced in 3D PF microcarriers (Cripto_(3D)_) or commercially available Cripto-1 purchased from R&D Systems (Cripto_(R&D)_). Two control groups were also examined, including myoblasts grown in starvation medium containing basic fibroblast growth factor (bFGF) as a proliferation inducer (positive control) and myoblasts grown in starvation medium with no supplements (negative control). The results confirmed that C2C12 proliferation was significantly increased in the presence of Cripto_(3D)_ compared with the negative control group. Myoblasts treated with Cripto_(R&D)_ or bFGF displayed a significantly higher level of BrdU incorporation when compared to the negative control group but lower levels of BrdU incorporation when compared to the Cripto_(3D)_ treatment (Figure 4A). BrdU incorporation results comparing Cripto_(3D)_ and Cripto_(R&D)_ to negative controls showed a dose-dependent increase in the proliferative response of C2C12 myoblasts to Cripto-1 (Figure 4B). Cell counting experiments quantifying the number of live cells after each treatment showed similar trends among the different treatment groups (Figure 4C).

**Figure 5:**
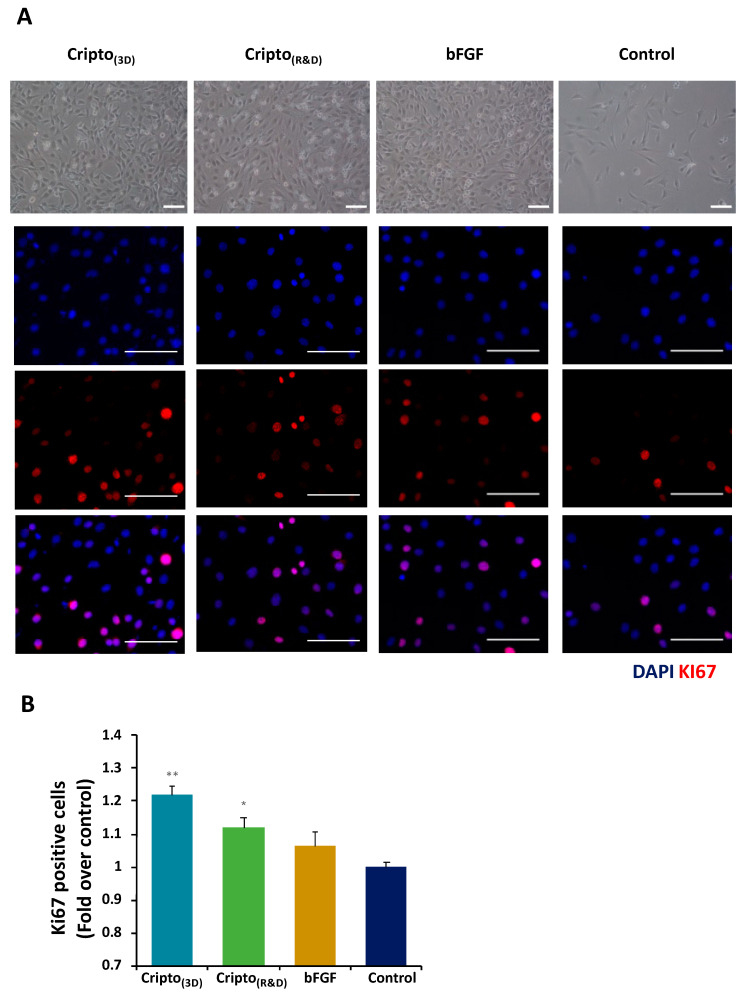
Proliferation analysis using Ki67 immunostaining of C2C12 myoblasts after treatment with Cripto produced in 3D microcarriers. C2C12 myoblast proliferation was evaluated by a Ki67 immunostaining assay after being cultured for 48 h in serum-free medium containing Cripto produced in 3D microcarriers (Cripto_(3D)_) or commercially available Cripto (Cripto_(R&D)_). Also evaluated were a bFGF medium positive control and a serum-free medium negative control. (**A**) Representative bright field images of myoblasts showing the morphology of the cells in the different treatments. Fluorescence staining for the cell proliferation marker Ki-67 (red) is shown for the different treatments. Scale bar 100 µm. (**B**) Ki67 levels were quantified and presented as fold change over control (serum-free medium). The proliferative effect of Cripto_(3D)_ was compared with that of commercial Cripto_(R&D)_. Results are shown as mean ± S.D. of at least three independent experiments. * indicates *p* < 0.05, ** indicates *p* < 0.01.

**Figure 6:**
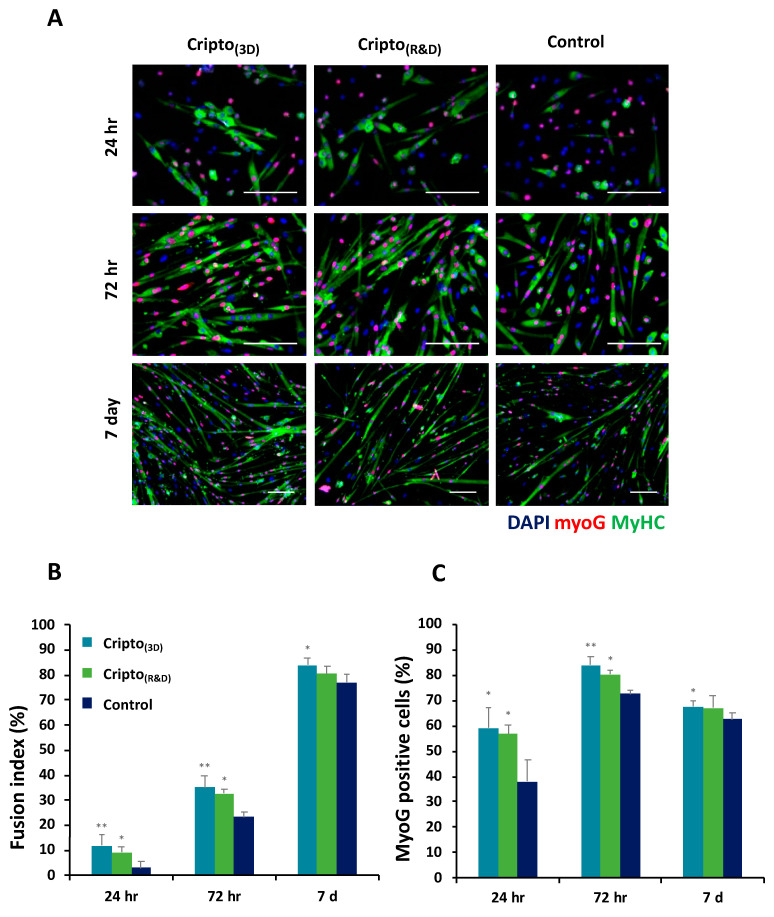
Cripto protein produced in 3D microcarriers retains the ability to induce muscle satellite cell commitment toward a differentiative fate. Muscle satellite cells were cultured in 10% HS medium containing Cripto_(3D)_ or commercial Cripto_(R&D)_ and compared to the control group (containing only 10%FBS medium). (**A**) Cells were stained with DAPI (blue), MyHC (green), and myoG (red), and representative images were obtained for 24 h, 72 h, and 7 days in culture. (**B**) Differentiation was estimated by measuring the fusion index, which is the percentage of nuclei within MyHC-positive cells. (**C**) Quantification of MyoG-positive nuclei per total myonuclei, as identified with a DAPI counterstain. Results are shown as mean ± S.D. of at least three independent experiments. * indicates *p* < 0.05, ** indicates *p* < 0.01.

The biological activity of purified Cripto-1 was investigated by staining the treated myoblasts with the proliferation marker Ki67. Bright field and fluorescence images of the cells were acquired and used to assess the effects on the cells. The bright field images showed that cells treated with Cripto_(3D)_ were more confluent than the untreated cells (Figure 5A). Fluorescence images were quantified for the percentage of Ki67-positive cells; the number of positive cells treated with Cripto_(3D)_ was the highest of all the treatments (Figure 5B). To further assess the bioactivity of the Cripto-1 produced in the PF microcarriers, we measured the effects of the protein on satellite cell differentiation. Satellite cells were grown in low-activation medium (10% HS) supplemented either with Cripto_(3D)_ or Cripto_(R&D)_ or without supplement as a negative control. Double immunostaining for the myogenic differentiation markers myosin heavy chain (MyHC) and myogenin (MyoG) was performed after 24, 72 h, and 7 days (Figure 6A). MyoG is a myogenic marker that is expressed during differentiation. High levels of this marker indicate that the differentiation capacity of the cells is elevated due to the presence of the Cripto proteins. These results are consistent with the previous findings of Guardiola et al.; they observed that treatment with Cripto increases the tendency of satellite cells toward differentiation and expression of MyoG [51]. The rates of myogenic differentiation were evaluated by measuring the fusion index, which is the percentage of nuclei within MyHC-positive myotubes (i.e., those with ≥2 nuclei) out of the total number of nuclei. Quantitative analysis of the images showed significantly increased fusion in cells treated with Cripto_(3D)_ compared to the negative controls at all time points (Figure 6B). The quantification of MyoG-positive cells at each time point also showed a significantly higher level of MyoG in cells treated with Cripto_(3D)_ compared to the negative control cells (*p* < 0.05, *n* ≥ 3) (Figure 6C). MyoG levels in cells treated with Cripto_(R&D)_ were also significantly higher compared to the negative control cells at the early time points (*p* < 0.05, *n* ≥ 3) (Figure 6C).

### 2.3. Stability and Binding Capacity of Recombinant Cripto during Long-Term Storage

The shelf life and stability of the recombinant Cripto_(3D)_ protein was evaluated for up to six years after production. After every manufacturing process, the batch of the purified Cripto-1 protein was snap-frozen in liquid nitrogen and stored at −80 °C. Protein samples from batches produced at different time points were thawed and quantified for their stability and integrity. The SDS-PAGE analysis results for Cripto-1 samples from different batches are shown in Supplementary Figure S7A. In all samples, the bands corresponding to Cripto-1 proteins that were stored for different periods showed little evidence of protein degradation that may have occurred during storage. These results are consistent with data obtained in a conducted stability study that used an ELISA to show that Cripto-1 protein retains its ability to bind AlK4 receptors when stored at −80 °C for up to six years (Supplementary Figure S7B).

### 2.4. Discussion

Recent clinical progress in novel protein therapeutics has drawn renewed interest in establishing more robust methods for producing complex recombinant proteins in suspension bioreactors, particularly with mammalian cell lines. These efforts involve a range of mammalian cell types being engineered to overexpress the therapeutic protein, which is either secreted directly into the culture medium or retained within cells and extracted afterwards [21,52,53,54]. In this study, our aim was to optimize and validate a PF hydrogel 3D microcarrier for the production of large quantities of Cripto-1 protein by employing Cripto-overexpressing HEK293 cells grown in suspension bioreactors. For this purpose, 3D microcarriers made from semisynthetic PF hydrogels were designed to provide both physical stability and bioactivity to enable HEK293 cell survival and proliferation within the microcarriers for up to 21 days in suspension culture. The ability of the cells to secrete large amounts of Cripto protein into the culture medium was an equally important feature of the PF microcarrier system that we sought to investigate.

Moving from 2D culture to 3D microporous hydrogel microcarriers is important when large amounts of therapeutic protein are required, such as in the case of Cripto [51,55,56]. However, there are some challenges when working with 3D hydrogel microcarriers. In particular, the composition of the microcarriers can affect cell survival and protein production. Studies have shown that mammalian cell viability, growth potential, and phenotype can be altered by adjusting the physical and biological features of the encapsulating hydrogel [43,57]. Premised on the concepts of controlling cell phenotype using these features, we first had to identify PF material properties that provided a good growth matrix for cultivating Cripto-overexpressing HEK293 cells in stirred suspension bioreactors. Specifically, the PEG polymer composition of the PF was adjusted to endow the hydrogel with sufficient mechanical strength to resist the shear forces associated with the stirred suspension bioreactors for a duration of up to 21 days without impeding the proliferation or survival of the cells. Three compositions of PF hydrogels were tested with HEK293 cells in 3D culture in a preliminary screening experiment, including a low modulus, intermediate modulus, and a high modulus formulation. These formulations all contained 8 mg/mL PF. Additional PEG-DA crosslinker (0–2% *w*/*v*) was added to the PF to increase the hydrogel modulus from G’ = 250 Pa to G’ = 1000 Pa and G’ = 2000 Pa (see Supplementary Figure S1). The preliminary screening experiment demonstrated that the modulus of the PF affected the proliferation of the HEK293 cells after 7 days in culture (Figure 2H). The higher modulus formulations appeared to reduce the proliferation of the HEK293 cells in the 3D culture. Additionally, of the three compositions that were tested, only the low modulus formulation was not stable in the bioreactor for the full 21 days. The intermediate and high modulus formulations were stable for 21 days; however, because the high modulus formulation impeded cell proliferation more than the intermediate modulus formulation (Figure 2G), we chose to continue all experiments using the intermediate modulus formulation.

The composition of the biological component in the PF hydrogel provided adequate bioactivity to allow for cell survival within the microcarrier matrix for the duration of the 21 days in suspension culture. The quantitative viability of the cells was measured using a trypan blue exclusion assay and a PI assay. Both techniques confirmed viability of greater than 80% throughout the culture period. These data are consistent with previous experiments using PF hydrogels to culture other cell types within the gels [42,43,58,59]. The UV photopolymerization in this study may have contributed to the loss of cell viability immediately after gel formation. We and others have verified the cytocompatibility of UV photopolymerization [60], but we cannot exclude the possibility that either the Irgacure2959 and/or the 365 nm UV light adversely affected the HEK293 cells.

In terms of cell morphology, the HEK293 cells inside the hydrogel were initially rounded when the hydrogel was formed and remained rounded throughout the duration of the culture. Although anchorage-dependent, the HEK293 cells do not appear to spread within the PF matrix upon their encapsulation. Typically, stromal cells cultured in PF hydrogels with similar properties exhibit morphogenesis, leading to cell spreading within the matrix. We have previously cultured fibroblasts in PF hydrogels with evidence of cell adhesion, including the formation of focal adhesions [61]. Therefore, we assume that cell adhesion is possible between the HEK293 cells and the PF matrix, although we did not evaluate the formation of focal adhesions in this study. Cell morphogenesis may also be influenced by the ability of the cells to proteolytically break down the PF matrix. In hydrogels that are more highly crosslinked with additional PEG-DA, this proteolysis can be hampered [62]. In this study, we cultured the HEK293 cells in PF hydrogels, with slow proteolytic degradation owing to the additional PEG-DA that was added [61]. Hence, the HEK293 cells growing in the PF material that supports cell adhesion may more slowly degrade PF, which can explain why they appear to have a rounded morphology. This PF formulation was chosen to prevent the hydrogels from degrading prematurely during the culture period. Additionally, it is important to note that the HEK293 cells do not necessarily exhibit mesenchymal cell properties and may therefore have a limited ability to express a non-rounded cell morphology in 3D culture. This could be attributed to a limited production of proteases to break down the PF matrix, thus reducing their ability to form cellular extensions within the PF hydrogel milieu.

As a preliminary proof of concept with the 3D PF microcarriers, Cripto-1 production yields from 3.2 × 10^6^ HEK293 cells were measured at nearly 4.5 mg of Cripto after 21 days in culture. Although these values are not optimized, they represent an initial assessment of the potential yields achieved by this system. When compared to the cell-normalized yields obtained with conventional 2D tissue culture dishes (i.e., TCP), the 3D methods proved far superior. The cells in the 2D system approached a density threshold, which led to cell detachment from the plate, whereas cell proliferation in the 3D system was controlled by the mechanical properties of the PF microcarriers [21]. Although the 2D and 3D cultures can be further optimized, these results stand in agreement with previously reported data suggesting that cell proliferation is high and uncontrolled in a 2D system compared to the number of cells being maintained at a steady state in the 3D system [21]. More optimizations can also be performed on the 3D system, including optimization of the properties of PF for longer culture durations, optimization of growth/harvest cycle durations, changes in the type of bioreactor used, and control of bioreactor growth conditions using continuous monitoring for optimal production [9,63]. Consequently, the matrix modulus of G’ = 1000 Pa was chosen for the Cripto production experiments based, in part, on limiting cell growth within the matrix.

In addition to the high yields of 3D microcarrier Cripto-1 production, high-throughput purification is an important part of bioprocessing. Cripto-1 purification can become a bottleneck in cost-effective production of biotherapeutics. We addressed this issue using a purification strategy that is based on the creation of a fusion Cripto-1 protein with His-tag sequences that are biologically active [5,64] and which can be purified via affinity chromatography. This is a widely accepted strategy for the purification of recombinantly expressed clinically useful proteins [5,65,66,67]. Other techniques have been applied to recombinantly express and purify His-tag-fused Cripto-1 protein, particularly in non-mammalian expression systems such as *E. coli*. A recent study optimizing the production of recombinant soluble human Cripto-1 protein using the T7 expression system was performed by Senso et al. [67]. They found that a functional form of soluble Cripto-1 was difficult to obtain because of the 12 cysteine residues in the protein, which impairs the folding process. They developed a special purification process to obtain His-tagged Cripto-1 protein from inclusion bodies under denatured conditions [65]. The purification process included not only a Ni-NTA column step, but also a CDR-modified cellulose column to remove cellular debris. This step was required, in part, because the protein in *E. coli* is mostly confined to the inclusion bodies. However, this was not required in the HEK293 cells cultured within the 3D microcarriers as the protein is secreted, modified, and then collected directly from the medium. In general, the purification of Critpo-1 secreted outside the membrane is easier than the purification of proteins from cell lysates [5,68,69], underscoring the advantage of producing Cripto-1 with a HEK293 cell expression system in 3D microcarriers.

Beyond the use of affinity chromatography, we applied a couple of other well-established purification steps to achieve the high-quality, high-throughput recombinant protein yields. The first step involved concentrating the Cripto-1 protein in the collected culture medium via ultrafiltration to prepare the solution for chromatography. The second step involved the elution of the His-tagged Cripto-1 protein in a Ni-NTA column. The final step was to dialyze the purified Cripto-1 against PBS. The Ni-NTA affinity column demonstrated high capacity for loading the target protein, and no destructive effects were observed during the elution. Overall, the broad band at 27 kDa, which appeared in the first elution fraction, coupled with the absence of bands at 27 kDa in other fractions, confirms the high efficiency of the Cripto purification steps. Our study has also demonstrated that the amount of active protein increased when compared to 2D production of the protein. This suggests that both of the 3D production and purification processes were delicate enough to preserve the natural biological structure, which is strongly related to bioactive function.

In terms of measuring the bioactivity of Cripto-1, previous studies have shown that Cripto-1 plays a dual role by both increasing the proliferation rate of myoblasts and promoting satellite cells toward myogenic differentiation [51]. Cripto-1 binds Nodal and activates ALK4 signaling pathways under normal physiological conditions [70]. Hence, we characterized the activity of the expressed protein using a proliferation assay with the C2C12 myoblast line and a myogenic differentiation assay on primary myoblasts. The highest proliferation rate was found in myoblasts treated with Cripto_(3D)_ and Cripto_(R&D)_. In addition, a dose-dependent pattern of proliferative activity was shown in C2C12 cells exposed to Cripto_(3D)_ and Cripto_(R&D)_. These results were consistent with a previous in-depth study that demonstrated how Cripto-1 regulates muscle regeneration by attenuating the TGF-β ligand myostatin signaling pathway. According to this study, by antagonizing myostatin, Cripto-1 promotes myogenic cell proliferation, and by blocking myostatin activity, Cripto-1 increases the tendency of satellite cells to differentiate [51]. Based on these findings, we further investigated the effect of Cripto-1 on muscle satellite cell differentiation. The results here showed an enhanced differentiation rate for cells treated with Cripto_(3D)_ and Cripto_(R&D)_, which confirms the dual role of Cripto-1 as a regulator that increases the proliferation rate of myoblasts and promotes satellite cells toward myogenic differentiation. Not only was differentiation enhanced in the presence of Cripto-1 (both from 3D and R&D sources), but cell confluency in the differentiation assay was greater (the initial concentration of cells that were seeded was the same for all treatments). This result implies that the presence of Cripto affected proliferation as well as differentiation, although the enhanced differentiation could be also explained as a consequence of the increased proliferation (rather than a direct effect on differentiation). In the results of the bioactivity assays, a slight difference in bioactivity in favor of Cripto_(3D)_ was observed; however, this elevated bioactivity of Cripto_(3D)_ may be attributed to the fibrinogen residues from PF microcarriers that are not removed during purification steps (see Figure 3A). Future improvements to the purification process would thus be required to remove all fibrinogen fragments from the product so that Cripto_(3D)_ could be used in a clinical setting.

Storage stability is another important consideration when evaluating the commercial potential of a specific protein in the pharmaceutical market. A critical concern associated with therapeutic protein production is their ability to survive after the manufacturing process for long-term storage before clinical administration. As a result of extended storage durations, protein quality can be jeopardized by degradation and conformational changes [71,72]. The results presented in this work confirm that Cripto-1 protein produced using the 3D cultivation system is capable of maintaining its stability and integrity for at least 6 years. Data on protein characteristics obtained by SDS-PAGE and ELISA assays applied on samples of Cripto-1 batches from different time points show that protein stability and bioactivity were not affected by the long-term storage durations. Specifically, structural integrity was identified by the uniform bands in the SDS-PAGE results for samples from all time points, indicating that no protein degradation was detected in samples stored for up to 6 years. The ability of Cripto-1 to bind to the AlK4 receptor indicates that it retained its active conformation during long-term storage at −80 °C.

## 3. Conclusions

A 3D PF microcarrier-based cultivation system was designed for the production of Cripto-1 using HEK293 cell lines in stirred suspension bioreactors. The PF microcarrier maintained its mechanical integrity for up to 21 days in the stirred bioreactor. The mechanical properties of the PF did not prevent the growth of the HEK293 cells, nor did they obstruct the release of the constitutively expressed Cripto-1 protein from the microcarrier into the culture medium. The bioactivity of the purified Cripto-1 protein obtained from 3D PF microcarrier cultures was equivalent to commercially available Cripto-1, despite a near 10-fold increase in production yields obtained from the 3D system compared to conventional 2D production. The 3D PF microcarriers can thus help streamline the biomanufacturing of Cripto-1 in HEK293 cell lines and possibly improve production yields in other therapeutic proteins that require anchorage-dependent mammalian cell expression systems.

## 4. Materials and Methods

### 4.1. Synthesis of PEG-Diacrylate (PEG-DA) and PEG-Fibrinogen (PF)

PEG-diacrylate was synthesized as described elsewhere [44]. Briefly, linear PEG-OH with an average molecular weight of 10 kDa (Fluka, Buchs, Switzerland) was reacted with acryloyl chloride (Merck, Darmstadt, Germany) at a molar ratio of 1.5:1 relative to OH groups in dichloromethane (Aldrich, Sleaze, Germany) and triethylamine (Fluka, Buchs, Switzerland). The final product was precipitated in ice-cold diethyl ether (Frutarom, Haifa, Israel), followed by vacuum drying for 48 h. The degree of acrylation was quantified by NMR (nuclear magnetic resonance spectroscopy). PEGylated fibrinogen was prepared by conjugating PEG-DA, via Michael-type addition, with denatured, reduced fibrinogen chains according to previously described protocols [44,73]. Briefly, a 7 mg/mL solution of bovine fibrinogen (ID bio, Baixas, France) in 150 mM PBS containing 8 M urea was reacted with Tris(2-carboxyethyl) phosphine hydrochloride (TCEP–HCl) (Sigma–Aldrich). The molar ratio of TCEP–HCl to fibrinogen cysteines was 1.5:1. Once the protein was dissolved, PEG-DA in a solution of PBS and 8 M urea (280 mg/mL) was added at a molar ratio of 4:1, and the reaction was carried out for 3 h at room temperature in the dark. The PEGylated fibrinogen protein was then precipitated by adding 4 volumes of acetone (Bio-lab) and was re-dissolved in PBS-urea to the desired concentration, followed by dialysis (Spectrum 12–14 kDa MW Cutoff, USA) against 150 mM PBS for 24 h at 4 °C. Finally, the fibrinogen concentration in the product was measured by a NanoDrop spectrometer (A-280 nm, PF coefficiency-15.1) and the degree of PEG substitution was calculated according to published protocols [73]. Rheological parameters were calculated using a strain-rate-controlled shear rheometer (AR-G2, TA Instruments, New Castle, DE, USA) with a 20 mm parallel-plate geometry. Each measurement was carried out using 200 µL of hydrogel precursor solution containing 0.1% *w*/*v* Irgacure2959 photoinitiator. All rheological experiments were performed in triplicate. Time sweep oscillatory tests were conducted under a constant strain amplitude of 1% and a constant frequency of 2 Hz, which was determined to be in the linear viscoelastic region (LVR) of the PF hydrogels (data not shown). The shear storage and loss moduli (G’, G″) of the hydrogels was measured for each batch of PF materials (Supplementary Figure S1A) [43]. Increasing the concentration of additional PEG-DA was used to increase the G’, as described elsewhere (Supplementary Figure S1B,C) [33]. Three formulations of PF hydrogels were identified for screen testing, including a low modulus (G’ = 250 Pa), an intermediate modulus (G’ = 1000 Pa), and a high modulus (G’ = 2000 Pa) gel.

### 4.2. Cell Line Maintenance and Expansion in 2D Culture

Recombinant Cripto protein was produced in HEK293 cells as a Histidine-tagged (C-terminus 6xHis) fusion protein lacking the COOH-terminal amino acid residues +156 to +172 of Cripto (Minchiotti et al., 2011). Briefly, the Cripto-His (sequence from nucleotide −5 to +156 of the Cripto cDNA) expression vector was obtained via PCR using the complete Cripto cDNA as a template and the appropriate oligonucleotides [68]. The amplified fragment was cloned into a pcDNA3-His expression vector containing the 6XHis tag and the neomycin (geneticin) resistance gene for the selection of stable cell lines. HEK293 cells were transfected at 50% confluence by the calcium phosphate method using 10 μg of plasmid DNA; twenty-four hours after transfection, the cells were incubated with geneticin (G418)-containing medium for 3 weeks. Following the selection process, resistant cells were tested for recombinant Cripto-1 protein production via Western blotting [74]. Cripto-His is released to the medium as it lacks the COOH-terminal hydrophobic lipid anchor (GPI-anchor) [5]. Cell culture was performed in humidified incubators at 37 °C and in a HEPA-filtered atmosphere of air and 5% CO_2_. Cells were stored frozen in liquid nitrogen until use. At the onset of each experiment, the frozen cells were thawed and 4 × 10^6^ cells were seeded on round tissue culture plates (150 mm diameter × 20 mm height) in growth medium (ScienceCell) supplemented with 10% fetal bovine serum (FBS) (Biological Industries, Haemek, Israel) and 1% penicillin–streptomycin–ampicillin (Biological Industries, Israel). After 24 h of incubation, growth medium was changed to selection medium containing 200 µg/mL G418 (Gibco, Grand Island, NY, USA). Cells were split and expanded for 10–12 days in selection medium for the harvesting of large amounts of cells prior to cultivation in suspension bioreactors.

### 4.3. Cell Seeding in 3D PF Microcarriers

Cripto-overexpressing HEK293 cell lines were harvested from 40 tissue culture polystyrene (TCP) dishes at ~80% confluence by repeated pipetting to detach them from the surface. The suspended cells were then centrifuged for 2–5 min at 300× *g* to obtain a cell pellet. The pellet of cells was suspended with the PF hydrogel solution until there was no aggregation in the solution. Droplets of PF precursor solution with cells (7.5 × 10^6^–10 × 10^6^ cells/mL) were introduced on a superhydrophobic surface (fumed silica-coated glass plates prepared as described elsewhere [20,33]) using a 23-gauge syringe needle. The droplets of cells in hydrogel precursor were crosslinked under long-wave UV light (356 nm, 4–5 mW/cm^2^) for 1.5 min in the presence of 0.1% *w*/*v* of a photoinitiator (Irgacure^®^2959, Ciba, Basel, Switzerland) and then washed with growth medium into a 500 mL DURAN^®^ GL 45 bottle. The estimated volume of each bead (radius = 1.25 mm) was approximately 0.008 mL; therefore, there were approximately 6 × 10^4^ to 8 × 10^4^ cells per microcarrier.

### 4.4. Cell Viability and Imaging

The viability of the cells in the PF microcarriers was assessed qualitatively by fluorescence imaging using a calcein/ethidium assay. The cells in the microcarriers were stained with 4 mM calcein acetoxymethyl and 2 mM ethidium homodimer-1 (EthD). Calcein penetrates the cell membrane and emits a green fluorescence signal under the enzymatic activity of esterase (emission maximum at 515 nm). This green signal indicates the cells are alive. In contrast, EthD can only penetrate through the disrupted membranes of dead or dying cells. EthD attaches to the nucleic DNA of dying/dead cells and emits a red fluorescence signal at 620 nm. The cells seeded in 3D (PF microcarrier) were stained for 50 min on an orbital shaker at 37 °C with 5% CO_2_ and were washed with PBS at a ratio of 1:1. Stained constructs were visualized using a Nikon (TS100) fluorescence microscope with a ×2 or ×10 objective and imaged with a Nikon (DS-Fi1) camera. The quantitative viability of the encapsulated HEK293 cells was evaluated using the trypan blue exclusion assay and propidium iodide (PI) assay. PF microcarriers were washed with PBS and incubated at 37 °C with collagenase (0.5–1 mg/mL, Sigma) for 15–60 min to dissolve the 3D gel phase. After dissolution, the solution passed through a filter (70–100 μm) to dispose of large aggregates of cells and clumps of PF gel phase residues. The remaining pellet of cells was suspended with PBS to obtain the desired cell concentration of 5 × 10^5^ to 2 × 10^6^ cells per 1 mL PBS.

For the trypan blue assay, we utilized the fact that the cell membrane is selective to the entry of trypan blue; thus, penetrating and staining of the cells measures only the dead cell population. A volume of 10 μL containing a ratio of 1:1 PBS/cells and trypan blue (0.4% *w*/*v*) was loaded in an automated cell counter (Countess^®^-Invitrogen). The obtained results show a calculation of the number of cells, the percentage of living and dead cells, and the distribution of cell size. An example of this analysis data is provided in Supplementary Figure S2. For the PI assay, PI penetrates only dead cells, and once the dye is bound to nucleic acids, its fluorescence is enhanced up to 20- to 30-fold. The cells were removed from the microcarriers as described above and suspended in 1 mL PBS for PI staining. The staining solution was prepared by mixing 40 μg/mL PI reagent (Sigma) for DNA staining and 100 μg/mL RNases to exclude the staining of nucleic acids derived from RNA. After 10 min of incubation at 37 °C, cells were kept on ice to reduce the ongoing process of apoptosis. All samples were measured by flow cytometry (LSR- II, BD Biosciences). A positive control for dead cells was prepared using PI staining with 70% ethanol (Supplementary Figure S3). Blank samples of unstained cells were used as negative controls (Supplementary Figure S4). The gating strategy involved the use of positive and negative controls against the live cell population. The percentage of viable cells was calculated from the number of PI-positive cells in the entire population. The data were analyzed using a program called FCS Express 4 Plus Research Edition [75]. An example of these analysis data is provided in Supplementary Figures S3 and S4.

For microscopic evaluation of the HEK293 cells within the PF microcarriers, the cells were fixed in 10% formalin in PBS (Sigma) and stained for filamentous actin (f-actin) using a TRITC-phalloidin or FITC-phalloidin stain (sigma) and a nuclear stain, DAPI (sigma) or SYTOX-green (Thermo-Fisher), with both procedures performed according to the manufacturer’s recommendations. Briefly, the cells were permeabilized with 0.1% Triton X-100 in PBS (Sigma) for 10 min. The cells were then stained with FITC- or TRITC-labeled phalloidin (FTIC-phalloidin or TRITC-phalloidin, 1 μg/mL, Sigma) for 1 h at room temperature and counterstained through the addition of DAPI or SYTOX-green for 30 min. The cells were then washed three times with PBS at room temperature and then left overnight at 4 °C. The stained cells were imaged via confocal microscopy (Zeiss LSM700, Oberkochen, Germany) at a resolution of 1024 by 1024 pixels using a ×20 objective (numerical aperture = 0.45) and a z-step size of 2.3 μm per layer up to a depth of 200 μm.

### 4.5. Cultivation of Cells in Spinner Flasks

Cells in crosslinked PF microcarriers were cultured in suspension within GL 45 stirred reactors (DWK Life Sciences, Millville, NJ, USA) for up to 21 days. Typically, 12–15 mL of PF microcarrier was placed in 500 mL DURAN^®^ GL 45 bottles containing 200–300 mL of medium. The proportion of PF volume to culture medium volume in the bioreactor was normally 12 mL/280 mL. The estimated number of microcarriers in 280 mL of each bioreactor medium was approximately 1500 microcarriers, or 5 microcarriers in each ml of culture medium. The magnetic stirrer was set to 30–40 revolutions per minute (RPM). Two vents were used for air flow into the bioreactor. Increasing gas exchange in the medium was achieved with air pumps while keeping the conditions sterile using a 0.2 μm in-line pre-filter (Whatman PolyVENT™). The PF microcarriers containing HEK293 cells were subjected to a prescribed growth/starvation cycle for periodic harvesting of the secreted Cripto protein. Specifically, 3 days of growth medium was followed by 4 days of starvation/harvesting. Starvation medium included serum-free DMEM that was phenol-free and which contained 1% penicillin–streptomycin–ampicillin. After each 4-day starvation cycle, the medium was replaced with a fresh growth medium and incubated for an additional growth/harvesting cycle resulting in a total of three iterations (see Figure 1). This iterative cycling was chosen based on an initial optimization study evaluating cell viability in the microcarriers during long-term suspension culture in the bioreactors (data not shown). During the harvesting phase, the medium was collected every 24 h and further processed prior to protein purification. Briefly, cell debris was removed via centrifugation (10 min at 3000 RPM) and the harvested media was frozen at −80 °C until the beginning of the purification process.

### 4.6. Cripto Production in 2D Cultivation System

For the production of Cripto in a 2D culture system, the selection medium was removed from 40 TCP dishes and replaced with a starvation medium. After incubation at 37 °C and 5% CO_2_ overnight, the medium was collected and centrifuged. The harvested medium was stored at −80 °C prior to protein purification. The medium was then replaced with a fresh starvation medium, and these steps were repeated for three more days until the cells started to detach from the TCP surface.

### 4.7. Cripto Purification, Quantification, and Detection

Cripto protein was purified using a three-step procedure consisting of ultrafiltration, chromatography, and dialysis. A Centramate™ tangential flow filtration cassette membrane with a molecular weight cutoff of 10,000 Dalton was used for concentrating the Cripto from the collected culture medium (Pall Corporation, New York, NY, USA). This was followed by His-tag affinity chromatography with a Ni-NTA column (Qiagen, Hilden, Germany) to purify the Cripto. Finally, dialysis against PBS (130 mM) was conducted for 24 h at 4 °C, with four changes of the dialysis buffer performed to remove any impurities from the elution buffer. The purified Cripto solution was then frozen at −80 °C and freshly thawed for use at the beginning of each experiment. To qualitatively monitor purification efficiency, the Cripto protein fractions were withdrawn during the purification process and visualized by SDS-PAGE and Coomassie blue staining. Fractions were collected from the following steps: after concentration with ultrafiltration, flowthrough after the first and the second binding to the resin, each wash step, and from samples eluted from the Ni-NTA resin. To measure the concentrations of both the total purified protein and the active protein, an assay was carried out using a commercial ELISA kit (DuoSet, R&D systems, Minneapolis, Minnesota, United States). Serial dilutions of the concentrated protein were put into 96-well plates coated with either mouse Cripto antibody (for total concentration) or recombinant mouse activin receptor IB/Fc (for active protein concentration) (R&D systems AF1538 and 1477-AR, respectively). Briefly, 96–well plates were coated with 0.5 ng/mL of either activin RIB/ALK-4 (R&D systems 1477-AR) or Cripto antibody (R&D systems AF1538) in PBS (pH 7.5) overnight at 4 °C. After being washed three times, unbinding sites were blocked with 1% PBS-BSA for 2 h at room temperature (RT). The plates were then washed three times and Cripto samples were added (300 μg) and incubated for 2 h. The plates were incubated with 0.5 μg/mL His-tag biotinylated antibodies (R&D systems BAM 050) in PBS-Tween for 1 h at 37 °C and then for 1 h at RT. Finally, the plates were incubated for 1 h at RT with Streptavidin-HRP complex conjugated with HRP (R&D systems DY998). The plates were then developed with tetramethylbenzidine hydrogen peroxide substrate (R&D systems DY999), and absorbance was read at 450 nm on a Benchmark microplate reader (Bio-Rad Laboratories). Relative binding levels were determined by dividing the amount of Cripto that was bound to the activin RIB/ALK-4 antibody by the amount of Cripto that was bound to the Cripto antibody.

### 4.8. BrdU Incorporation Cell Proliferation Assay

A cell proliferation assay was used to measure the bioactivity of the recombinantly expressed Cripto, as has been more fully described elsewhere [51]. This assay was specially used to compare quantitative cell proliferation in the presence of Cripto produced using the PF microcarriers (Cripto_(3D)_) and that of commercially available Cripto purchased from R&D systems (Cripto_(R&D)_). Briefly, C2C12 skeletal muscle myoblasts were seeded on 96-well plates (10^4^ cells/well) in growth medium for 4 h. The growth medium was then replaced with starvation medium (DMEM, 0.5% FBS, 1% pen/strep) and incubated overnight in order to synchronize the mitotic cycle of the C2C12 cells. At the onset of the proliferation assay, the starvation medium was supplemented with either 500 ng/mL Cripto_(3D)_ or Cripto_(R&D)_. Two additional control groups were evaluated, including cells grown in starvation medium supplemented with basic FGF, as a positive control, and cells grown only in starvation medium, which was used as a negative control. For the dose–response assay, cells were treated with starvation medium containing either Cripto_(3D)_ or Cripto_(R&D)_ at increasing concentrations from 5 to 500 ng/mL. Cell proliferation was quantified using a BrdU Cell Proliferation Assay kit (Cell Signaling, 6813) according to the manufacturer’s instructions.

### 4.9. Ki67 Immunostaining Proliferation Assay

A Ki67 immunoassay was used to quantify cell proliferation in the presence of recombinantly expressed Cripto. Briefly, C2C12 cell viability was first quantified by staining cells with trypan blue (Biological Industries, Haemek, Israel) and measuring viability with an automated cell counter (Countess^®^ Invitrogen). The C2C12 myoblasts were then seeded on 24-well plates (3 × 10^4^ cells/well) and treated with starvation medium supplemented with either 500 ng/mL Cripto_(3D)_ or Cripto_(R&D)_. The positive control group included C2C12 cells grown in starvation medium supplemented with basic FGF, and the negative control group included C2C12 cells grown in starvation medium only. After 42 h in culture, the cells were fixed with 4% paraformaldehyde and permeabilized with 1% Triton X-100 in PBS. Cells were blocked in 5% BSA in PBS and incubated with rabbit polyclonal Ki-67 antibody (1:40, Abcam 15580) at 4 °C overnight. Subsequently, cells were incubated with donkey anti-rabbit AlexaFluor 555 (1:400; Invitrogen) and DAPI (1:1000; Invitrogen) at room temperature for 1 h. Finally, cells were mounted in mounting medium and visualized under a Zeiss LSM 700 confocal microscope (Carl Zeiss, Oberkochen, Germany). Bright field images were acquired using an inverted fluorescence microscope (Nikon Eclipse TS100, Nikon, Tokyo, Japan), a digital camera (Digital Sight, Nikon, Japan), and Nikon Nis-Elements F3.00 software (Nikon, Japan).

### 4.10. Muscle Satellite Cell Differentiation Assay

Muscle satellite cells were isolated from 6 muscles of adult mice according to published protocols [76,77]. The cells were plated at 1.9 × 10^4^ cells/well in 24-well gelatin-coated cell culture plates in BIO-AMF-2 medium (Biological Industries Ltd.). After 72 h, the medium was replaced with low-activation medium (DMEM/F12, 10% horse serum (HS), 1% pen/strep) and the cells were subjected to one of three different treatments: 500 ng/mL Cripto_(3D)_, 500 ng/mL Cripto_(R&D)_, or the control (low-activation medium only). The cells were incubated for 24 h, 72 h, and 7 days, fixed in 4% paraformaldehyde, and stained with DAPI, MyoG, and MyHC. All cells were imaged using a Zeiss LSM 700 confocal microscope (Carl Zeiss, Oberkochen, Germany). The percentage of MyoG-positive cells and MyHC-positive cells, as well as the fusion index, was measured and analyzed using Image-J software version 1.530.

### 4.11. Stability and Functionality of Cripto over Time

The stability and bioactivity of the Cripto_(3D)_ protein, taken from batches that were produced at different time points, was determined using SDS-PAGE and Coomassie blue staining, as well as ELISA assays. Briefly, samples of Cripto_(3D)_ that were stored for up to 6 years at −80 °C were loaded into NuPAGE Tris-base 10% gels (Life Technologies, Australia) at a loading concentration of 5µg protein per band, and SDS-PAGE was performed following the manufacturer’s protocol. The effect of shelf life on Cripto functionality was determined using an ELISA bioactivity kit (R&D systems, DuoSet) according to the manufacturer’s protocol, as described in detail above.

### 4.12. Statistical Analysis

All data were obtained from at least three independent experiments (*n* ≥ 3) and expressed as mean ± standard deviation (S.D.). Statistical analysis was performed using the un-paired Student’s *t*-test. In all experiments, significance is considered as follows: * *p* < 0.05, ** *p* < 0.01, *** *p* < 0.001, and **** *p* < 0.0001.

## Data Availability

The data presented in this study are available on request from the corresponding author.

## Supplementary Materials

The following supporting information can be downloaded at: https://www.mdpi.com/article/10.3390/gels9030243/s1, Figure S1: Rheological properties of the PEG-Fibrinogen (PF) hydrogels; Figure S2: Representative data from cell viability analysis using the Trypan blue exclusion assay; Figure S3: Representative data from cell viability analysis using the Propidium Iodide (PI) assay; Figure S4: Representative data from the Propidium Iodide (PI) assay; Figure S5: Representative confocal images of live/dead staining of microcarriers shown at high magnification.; Figure S6: Representative confocal image stacks of f-actin-stained colonies in the PF microcarriers shown at high magnification; Figure S7: Stability and bioactivity of recombinant Cripto produced in 3D microcarriers following long term storage.
