# Supplementary material for: Biomanufacturing Recombinantly Expressed Cripto-1 Protein in Anchorage-Dependent Mammalian Cells Growing in Suspension Bioreactors within a Three-Dimensional Hydrogel Microcarrier
Source: Gels. 2023 Mar 18;9(3):243. doi: 10.3390/gels9030243 (PMC10048735; doi:10.3390/gels9030243)
Supplement: Supplementary file 1 [file gels-09-00243-s001.zip › gels-2209480-supplementary.pptx]

## Slide 1
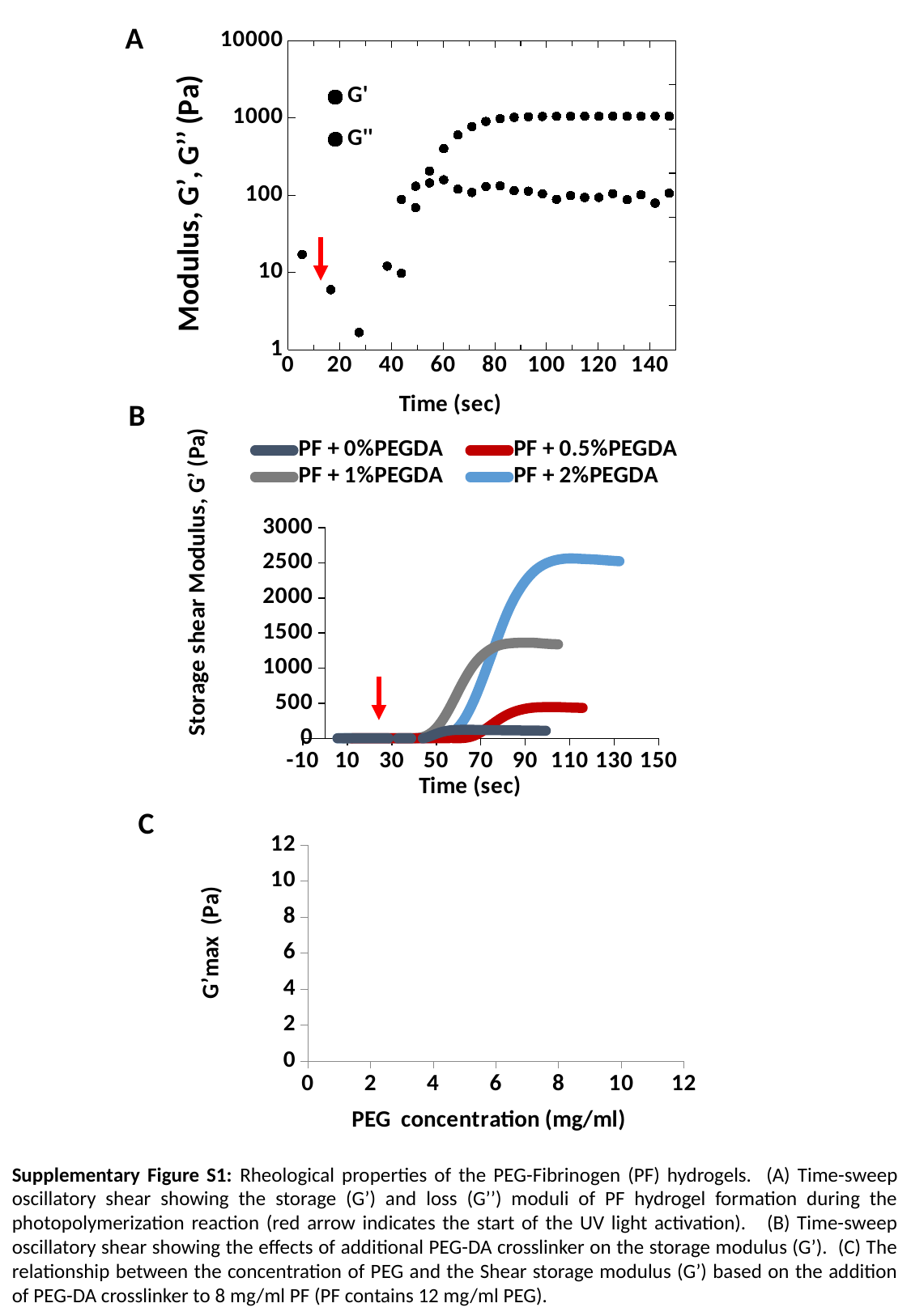

A
### Chart
| Category | G' | G'' |
|---|---|---|B
### Chart
| Category | PF + 0%PEGDA | PF + 0.5%PEGDA | PF + 1%PEGDA | PF + 2%PEGDA |
|---|---|---|---|---|C
### Chart
| Category | PEG-Fib |
|---|---|Supplementary Figure S1: Rheological properties of the PEG-Fibrinogen (PF) hydrogels. (A) Time-sweep oscillatory shear showing the storage (G’) and loss (G’’) moduli of PF hydrogel formation during the photopolymerization reaction (red arrow indicates the start of the UV light activation). (B) Time-sweep oscillatory shear showing the effects of additional PEG-DA crosslinker on the storage modulus (G’). (C) The relationship between the concentration of PEG and the Shear storage modulus (G’) based on the addition of PEG-DA crosslinker to 8 mg/ml PF (PF contains 12 mg/ml PEG).

## Slide 2
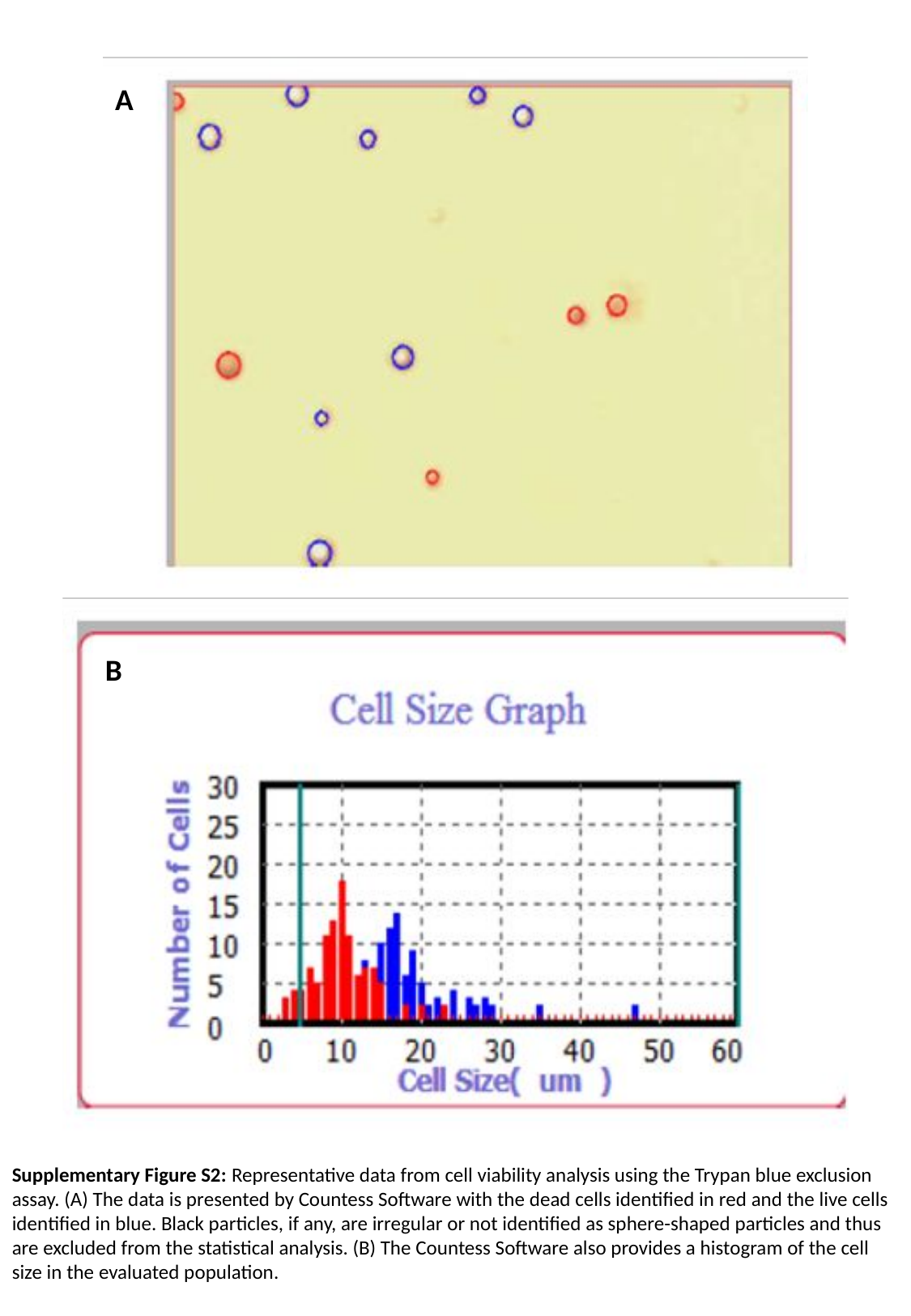

A
B
Supplementary Figure S2: Representative data from cell viability analysis using the Trypan blue exclusion assay. (A) The data is presented by Countess Software with the dead cells identified in red and the live cells identified in blue. Black particles, if any, are irregular or not identified as sphere-shaped particles and thus are excluded from the statistical analysis. (B) The Countess Software also provides a histogram of the cell size in the evaluated population.

## Slide 3
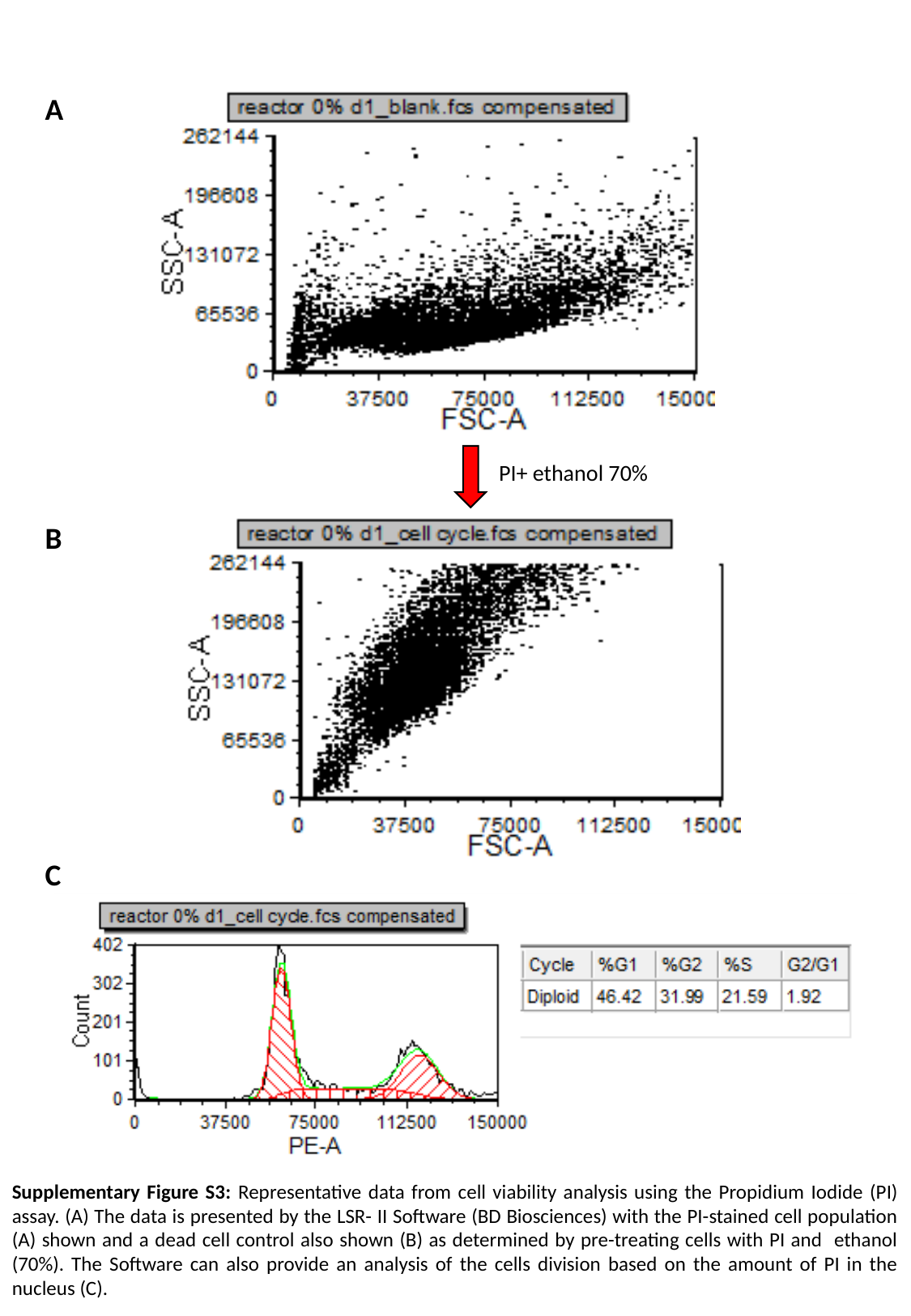

A
PI+ ethanol 70%
B
C
Supplementary Figure S3: Representative data from cell viability analysis using the Propidium Iodide (PI) assay. (A) The data is presented by the LSR- II Software (BD Biosciences) with the PI-stained cell population (A) shown and a dead cell control also shown (B) as determined by pre-treating cells with PI and ethanol (70%). The Software can also provide an analysis of the cells division based on the amount of PI in the nucleus (C).

## Slide 4
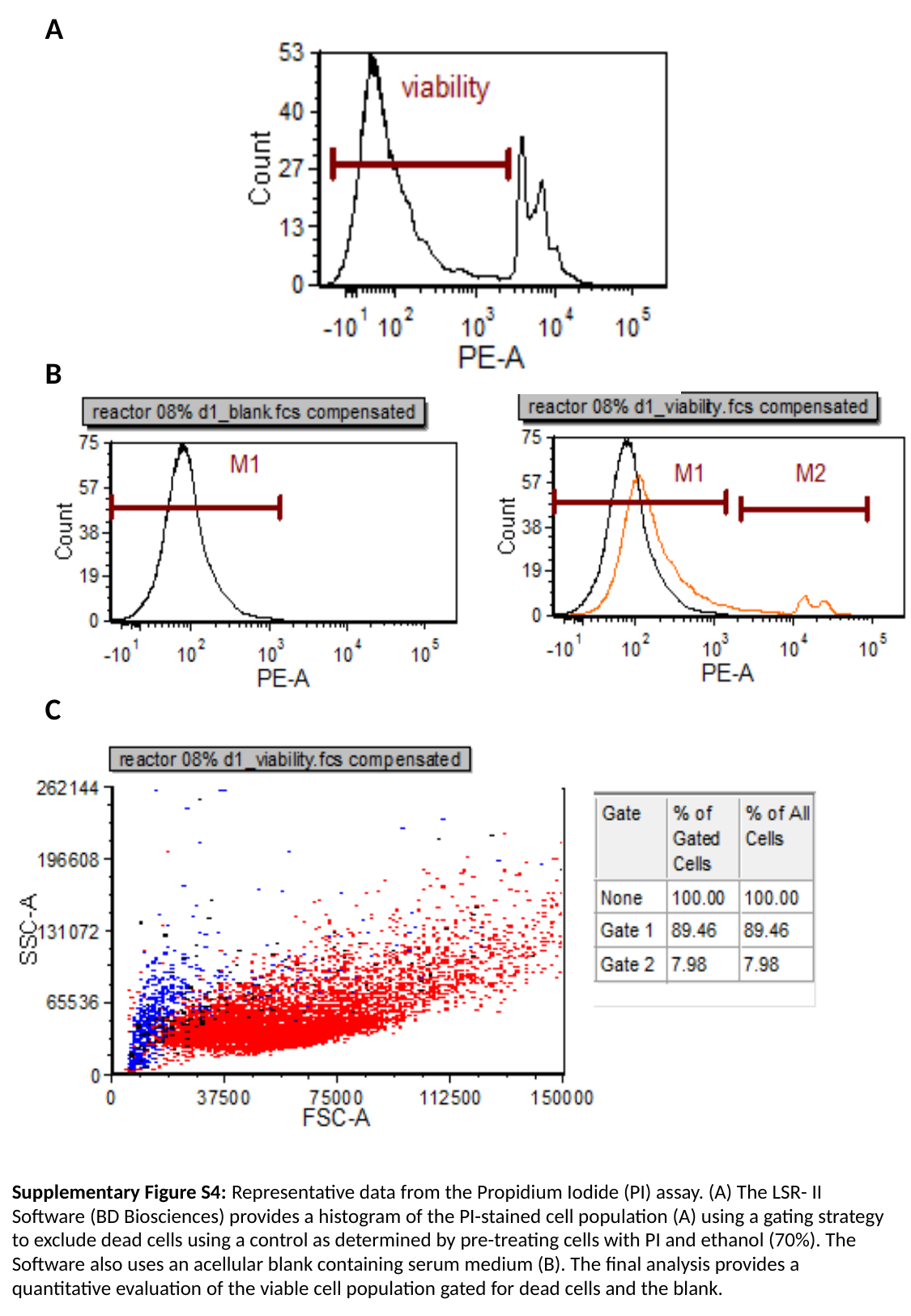

A
B
C
Supplementary Figure S4: Representative data from the Propidium Iodide (PI) assay. (A) The LSR- II Software (BD Biosciences) provides a histogram of the PI-stained cell population (A) using a gating strategy to exclude dead cells using a control as determined by pre-treating cells with PI and ethanol (70%). The Software also uses an acellular blank containing serum medium (B). The final analysis provides a quantitative evaluation of the viable cell population gated for dead cells and the blank.

## Slide 5
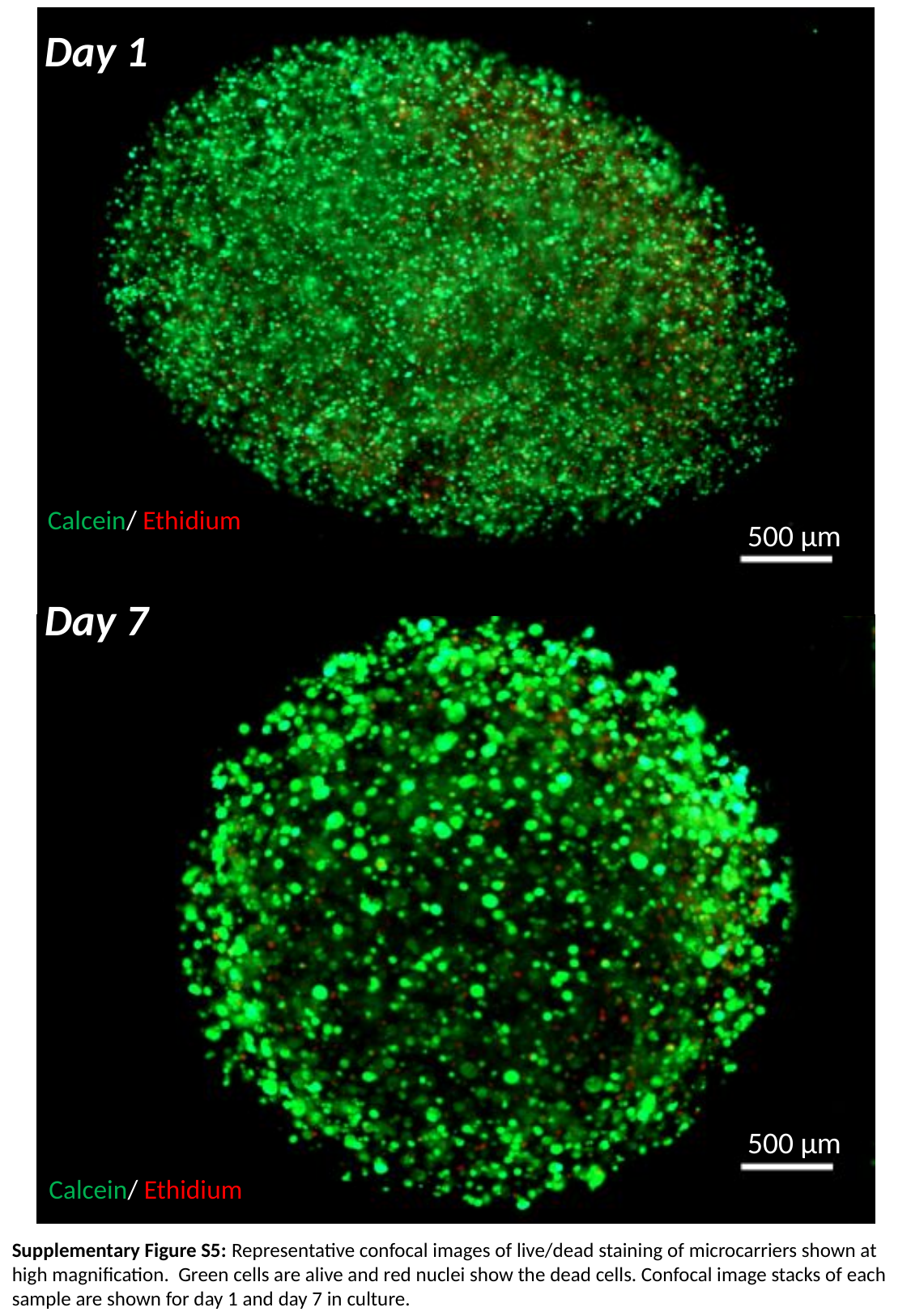

Day 1
Calcein/ Ethidium
500 µm
Day 7
500 µm
Calcein/ Ethidium
Supplementary Figure S5: Representative confocal images of live/dead staining of microcarriers shown at high magnification. Green cells are alive and red nuclei show the dead cells. Confocal image stacks of each sample are shown for day 1 and day 7 in culture.

## Slide 6
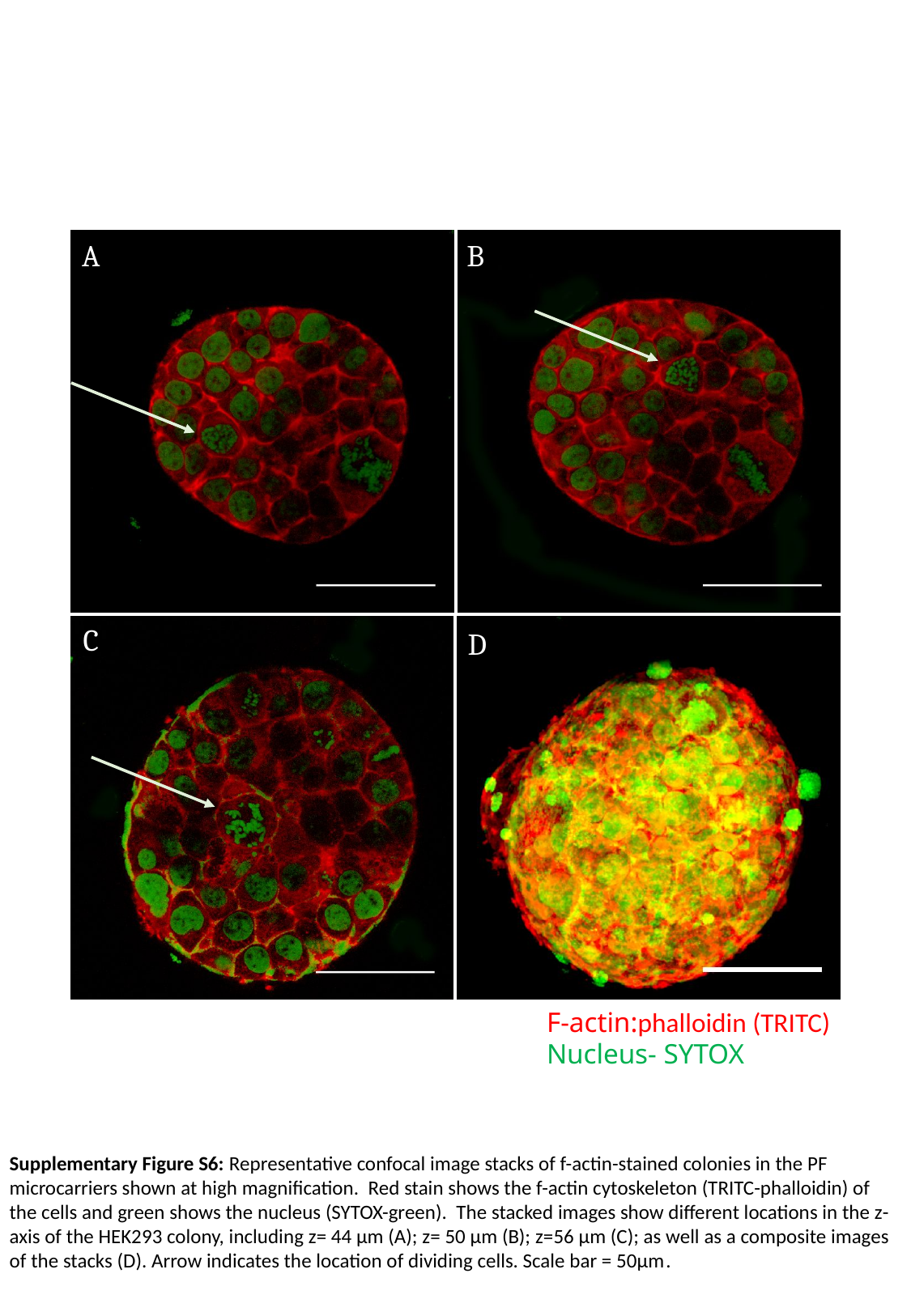

A
B
C
D
F-actin:phalloidin (TRITC)
Nucleus- SYTOX
Supplementary Figure S6: Representative confocal image stacks of f-actin-stained colonies in the PF microcarriers shown at high magnification. Red stain shows the f-actin cytoskeleton (TRITC-phalloidin) of the cells and green shows the nucleus (SYTOX-green). The stacked images show different locations in the z-axis of the HEK293 colony, including z= 44 µm (A); z= 50 µm (B); z=56 µm (C); as well as a composite images of the stacks (D). Arrow indicates the location of dividing cells. Scale bar = 50µm.

## Slide 7
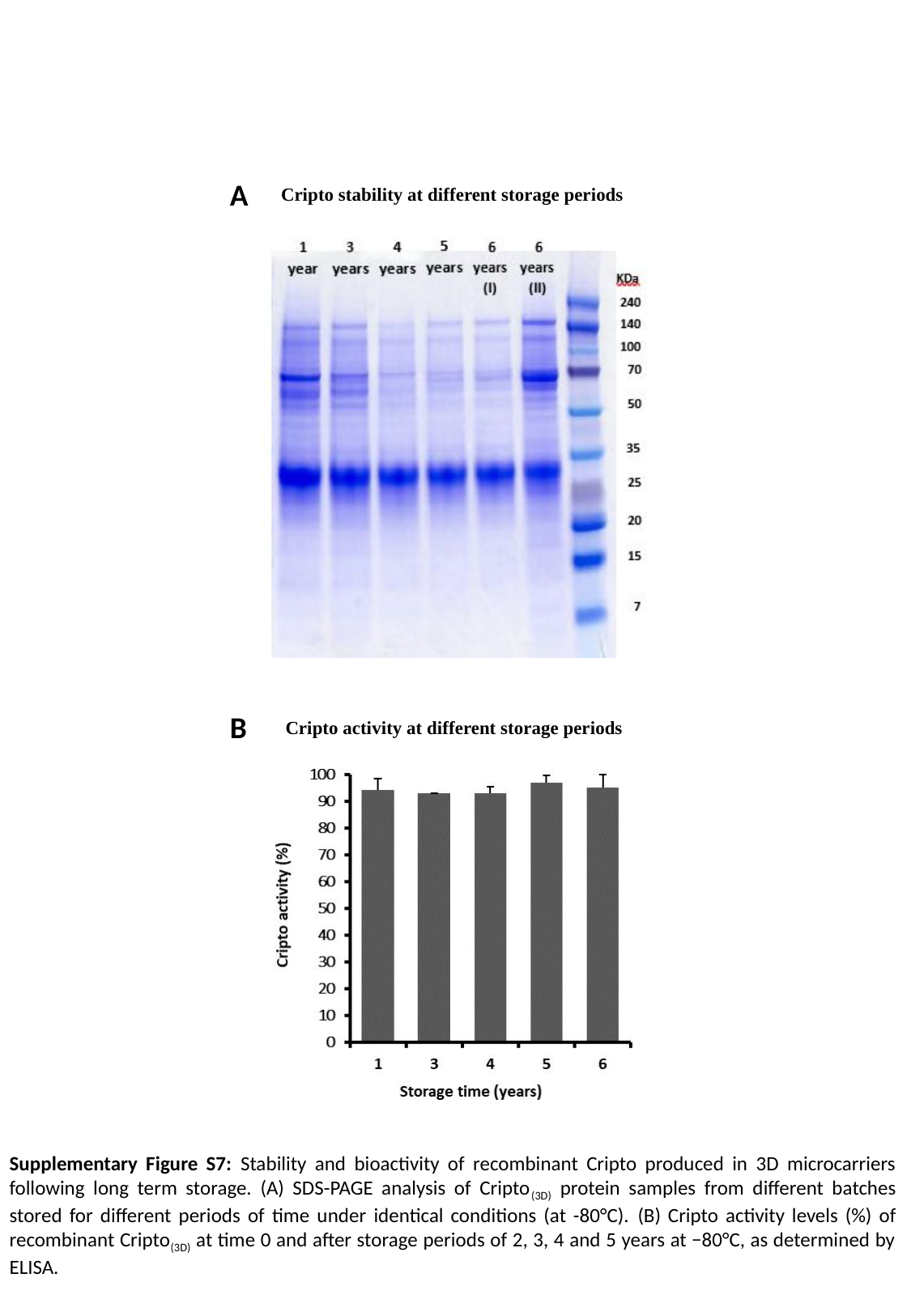

A
Cripto stability at different storage periods
B
Cripto activity at different storage periods
Supplementary Figure S7: Stability and bioactivity of recombinant Cripto produced in 3D microcarriers following long term storage. (A) SDS-PAGE analysis of Cripto(3D) protein samples from different batches stored for different periods of time under identical conditions (at -80°C). (B) Cripto activity levels (%) of recombinant Cripto(3D) at time 0 and after storage periods of 2, 3, 4 and 5 years at −80°C, as determined by ELISA.
